# Assessing left ventricular systolic function in children with a history of Kawasaki disease

**DOI:** 10.1186/s12872-020-01409-0

**Published:** 2020-03-12

**Authors:** Zhou Lin, Jingjing Zheng, Weiling Chen, Tingting Ding, Wei Yu, Bei Xia

**Affiliations:** Department of Ultrasound, Shenzhen Children’s Hospital, Shenzhen, China

**Keywords:** Kawasaki disease, Speckle tacking echocardiography, Long-term, Follow-up

## Abstract

**Background:**

The incidence of Kawasaki disease (KD) is increasing. Indeed, KD has become the most common cause of acquired heart disease in children. Previous studies have well summarized the acute phase left ventricular (LV) systolic dysfunction using speckle tracking echocardiography (STE); however, changes in LV systolic function after long-term follow-up remain unclear.

**Methods:**

One hundred children with a history of KD, but without coronary artery aneurysms, were enrolled. These children were divided into two subgroups based on the presence or absence of coronary artery dilatation (CAD). The duration of follow-up was > 7 years. The control group consisted of 51 healthy children. The LV myocardial strain were measured by two- and three-dimensional STE.

**Results:**

Two-dimensional STE not only revealed that LV longitudinal strain decreased in part of segments in both KD groups, but also showed that global strain decreased in the KD group with CAD compared to the controls (*P* < 0.05). Global longitudinal strain (GLS), global circumferential strain (GCS), global radial strain (GRS), and global area strain (GAS) were obtained by 3D STE. Compared to the controls, GLS and GAS decreased in both KD groups (*P* < 0.05). GCS and GRS decreased in the KD group with CAD, but was unchanged in the KD group without CAD (*P* < 0.05).

**Conclusions:**

LV systolic dysfunction in children with KD and CAD was more severe than KD children without CAD compared to healthy children. This dysfunction can be assessed by LV regional and global myocardial strain using two- and three-dimensional STE.

## Background

Kawasaki disease (KD) is an acute self-limited vasculitis that affects children, 85% of whom are < 5 years of age [1]. The incidence of KD has increased in the last three decades [2], making KD the most common cause of acquired heart disease in children [3]. KD mainly affects small- and medium-sized arteries, especially coronary artery. KD causes coronary artery lesions (CALs), including coronary artery dilatation (CAD) and coronary artery aneurysms [2].

The American Heart Association (AHA) algorithm, which was designed to improve the diagnosis for KD uses echocardiographic evidence of decreased left ventricular (LV) systolic function as supporting criteria [4]. The LV ejection fraction (LVEF) is the most commonly used conventional parameter to quantify global LV systolic function in children with KD; however, previous studies have usually demonstrated normal LVEF in the acute phase [5, 6]. Speckle tracking echocardiography (STE) has recently been shown to be a reliable method with which to assess LV systolic function and deformation through LV myocardial strain [7–9].

Since 2010 several studies have revealed impaired LV myocardial strain among children with KD in the acute phase using STE [5, 6, 8, 10]. Although myocardial function and coronary artery diameter in most patients have recovered after treatment [6, 8], there is evidence of ongoing myocardial and coronary arterial structural alterations in the long-term [11–13]. Few studies have focused on LV myocardial strain of children with a history of KD after long-term follow-up using STE, especially children with KD but without CAD [7–9]. Excluding coronary artery aneurysms, whether or not there is a long-term effect of CAD on LV myocardial strain is unclear. In this study we assessed LV myocardial strain of children with a history of KD, and with or without CAD, using two- and three-dimensional (2D and 3D) STE.

## Methods

### Subjects

Between February 2017 and February 2018, 100 children with KD (70 males and 30 females; mean age, 140.77 ± 25.48 month; age range, 90–215 months) were enrolled in this study for evaluation of LV myocardial strain at Shenzhen Children’s Hospital. All participants had a history of KD and fulfilled the diagnostic criteria for KD [4]. Participants with KD and coronary artery aneurysms were excluded (Aneurysms were defined by a Z-score ≥ 2.5). According to AHA guidelines, the participants were divided into two subgroups based on the presence of CAD. Patients without CAD were defined by a Z-score always < 2, while patients with CAD were defined by a Z-score of 2 to < 2.5 [14]. The interval from the onset to the start of this study was > 7 years. All participants received standard treatment and no participants had intra-venous immunoglobulin (IVIG) resistance. The control group consisted of 51 age- and gender-matched healthy children who underwent echocardiography for evaluation of a cardiac murmur during school. Based on echocardiographic, electrocardiogram, and myocardial enzyme biochemical testing, the 51 children in the control group were all considered healthy.

### Echocardiography examination

Echocardiographic evaluation was performed in the left lateral recumbent position using a Vivid E9 (GE Healthcare, Horten, Norway) with M5S and 4-V phased-array matrix transducers. All images and measurements were obtained from standard views according to the recommendations of the American Society of Echocardiography for chamber quantification. All images and datasets were digitally stored.

Routine 2D echocardiographic images were obtained in the parasternal and LV apical views. The LV end-diastolic dimension and left atrial anteroposterior dimension were obtained in the parasternal long-axis view. The LA boundary was delineated manually in the apical long-axis, four-chamber, and two-chamber views using customized software (details below). The software automatically measured the left atrial maximum volume (LAV_max_) and left atrial minimum volume (LAV_min_) [15]. LV end-diastolic volume, LV end-systolic volume, stroke volume, and LVEF were obtained using the biplane modified Simpson’s method. The peaks of early velocity (E wave) and late diastolic velocity (A wave) across the mitral valve were obtained. The ratio between the E and A waves (E/A) was calculated.

Tissue Doppler imaging was performed at the base of interventricular septum (septal parameters), at the lateral wall of the LV (lateral parameters) and at the anterolateral wall of the RV. Gain was minimized to obtain clear signals, and images were recorded at 100 mm/s. Myocardial velocity during systole (s’), early diastole (e’), and late diastole (a’) were measured [8].

The mitral annular plane systolic excursion (MAPSE) and tricuspid annular plane systolic excursion (TAPSE) were measured by two-dimensional echocardiography–guided M-mode recordings from the apical 4-chamber view as previously recommended [16–18].

### Two-dimensional STE longitudinal strain

Two-dimensional echocardiographic images of three cardiac cycles were obtained at a frame rate of 60–90 frames per second from the LV apical long-axis, four-chamber, and two-chamber views. The images were analyzed offline using customized software (EchoPAC V113; GE Healthcare). The LV endocardial boundary was manually delineated; the software automatically drew the LV epicardial boundary. The width of the region of interest was manually adjusted when necessary. The software automatically divided the LV myocardium into 17 segments, then generated curves for LV longitudinal strain [19].

The peak systolic strain was defined as the maximum value during the LV systolic phase. The regional longitudinal peak systolic strain was obtained in all 17 segments. In the LV apical long-axis, four-chamber, and two-chamber views, three types of global longitudinal strain (GLS) were obtained (GLS_LAX, GLS_A4C, and GLS_A2C) [6]. The software exported an averaged global LV myocardial strain, including all 17 segments and designated 2D GLS.

### Global 3D STE strain

Using a phased-array matrix transducer, the LV apical long-axis, four-chamber, and two-chamber views were demonstrated in the 4D mode, then “Large mode” was selected. The entire LV was depicted in the screen. The highest frame rate as possible (> 25 frames/sec) was acquired. The next step involved entering the “full volume mode”, acquiring six cardiac cycles sub-volumes to generate the pyramidal full-volume data set.

**Fig. 1:**
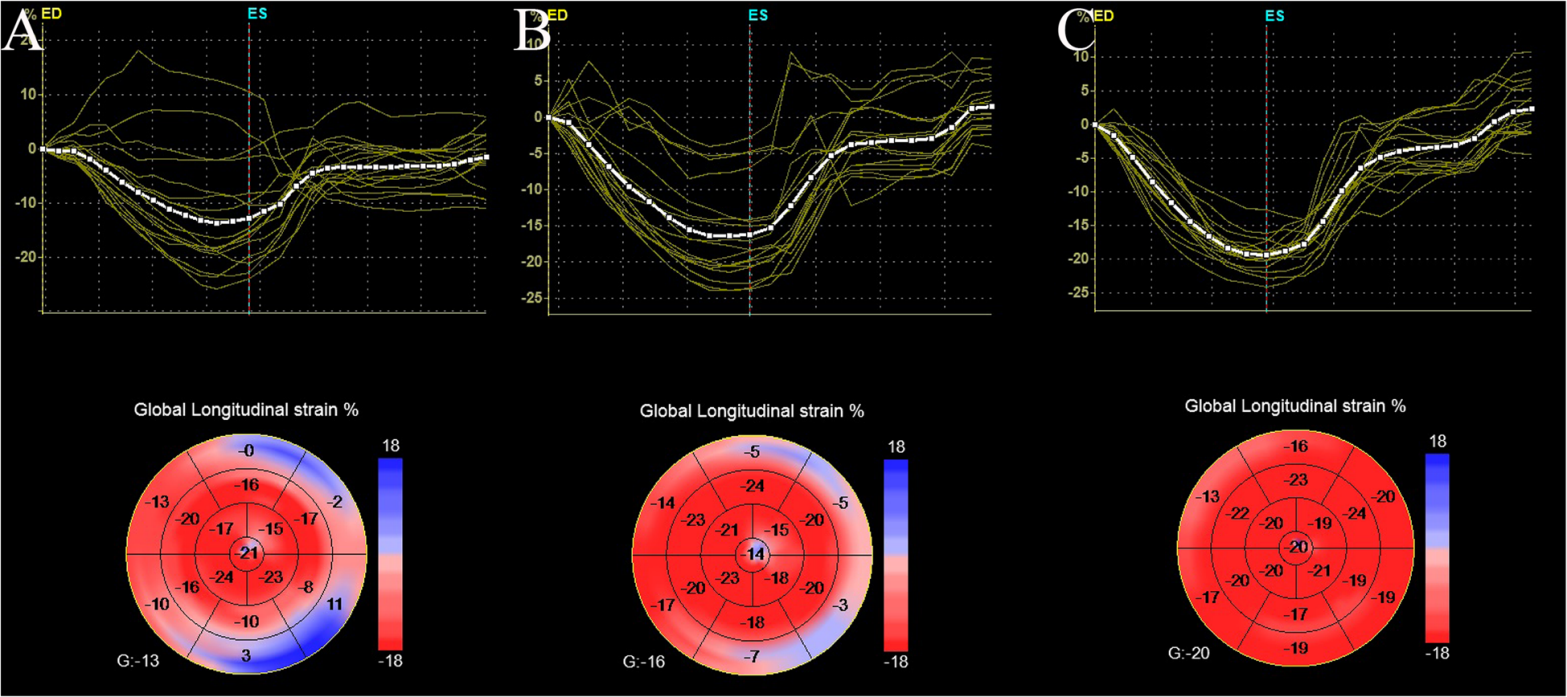
The difference of GLS between the KD with CAD group, KD without CAD group and the control group. **a** KD with CAD patient. **b** KD without CAD patient. **c** normal children. CAD: coronary artery dilation; GLS: global longitudinal strain; KD: Kawasaki disease

Four different components of global strain (global longitudinal strain [GLS] (Fig. 1), global circumferential strain [GCS], global radial strain [GRS], and global area strain [GAS]) were obtained by 3D STE. Customized software was used to automate the definition of the LV endocardial boundary with manual adjustment if necessary [20].

### Reproducibility

Ten subjects were randomly selected from each KD group to assess inter- and intra-observer agreement of 3D strain analysis for GLS, GCS, GRS and GAS, blinded to the previous results and using new arbitrary images. For the inter-observer variability assessment, the first observer performed the analyses. The second observer repeated the analyses within 24 h. For assessment of intra-observer variability, the analyses were repeated twice by the first observer within 1 week. The reproducibility results were analyzed using a coefficient of variation and standard errors of the mean and intraclass correlation coefficients with 95% confidence intervals.

### Statistical analysis

Continuous parameters are expressed as the mean ± standard deviation. Categorial parameters are expressed as frequencies. Differences in continuous parameters among the three groups were analyzed using one-way analysis of variance. Differences in continuous parameters between the two groups were analyzed using independent-sample *t*-tests. Differences in categorial parameters between the two groups were analyzed using Fisher’s exact test. General linear models were used to estimate the correlation between the altered segments of 2D region strain and the site of CAD. To assess the ability of 2D GLS, 3D GLS, 3D GCS, 3D GRS and 3D GAS, receiver operating characteristic (ROC) curves were computed and the area under the curve (AUC) was obtained. A two-tailed *P* < 0.05 was used to define statistical significance. Statistical analysis was performed using SPSS version 17.0 software.

## Results

### Clinical features

One hundred children with a history of KD and 51 healthy children were enrolled (Table 1). In the group of KD with CAD, 76% (41/54) were male, compared with 63% in the KD without CAD group and 67% in the control group. The differences among the groups with respect to age, heart rate, systolic blood pressure, diastolic blood pressure, body surface area, interval from onset-to-exam, interval from onset-to-IVIG treatment, and treatment strategy of using cardiovascular drugs (including three strategies: “Aspirin is administered at 80 to 100 mg/kg per day in 4 doses with IVIG”, “low-dose aspirin (3 to 5 mg/kg per day)”, and “2 to 6 mg/kg per day in 3 divided doses” [4], details are shown in Table 1) were not statistically significant (*P* > 0.05). The complete KD accounted for 33% in the KD with CAD group, compared with 46% in the KD without CAD group.

**Table 1:** Clinical features of three groups

	KD with CAD (*n* = 54)	KD without CAD (*n* = 46)	Controls (*n* = 51)	*P*
Male	41 (76%)	29 (63%)	34 (67%)	0.350
Age (months)	139.44 ± 25.27	144.41 ± 26.98	138.90 ± 24.46	0.510
Heart rate (bmp)	78.83 ± 10.59	75.56 ± 10.63	74.37 ± 14.50	0.150
SBP (mmHg)	108.20 ± 13.77	111.59 ± 12.92	110.63 ± 10.63	0.375
DBP (mmHg)	60.96 ± 11.59	61.09 ± 13.89	64.74 ± 9.76	0.188
BSA (m^2^)	1.35 ± 0.23	1.41 ± 0.22	1.34 ± 0.22	0.323
Interval from onset to exam (months)	116.83 ± 18.04	118.00 ± 20.93	/	0.765
Interval from onset to IVIG treatment (days)	10.36 ± 4.76	8.53 ± 3.34	/	0.145
Treatment strategy of using cardiovascular drug (1^a^/2^b^)	5/49	4/42	/	0.922
Dipyridamole^c^	23/54	24/46	/	0.339
Complete KD	18 (33%)	21 (46%)	/	0.208

*BSA* body surface area; *CAD* coronary artery dilation; *DBP* diastolic blood pressure; *IVIG* intravenous immunoglobulin; *KD* Kawasaki disease; *SBP* systolic blood pressure

^a^: Aspirin is administered at 80 to 100 mg/kg per day in 4 doses with IVIG, continue high dose aspirin until day 14 of illness and 48 to 72 h after fever cessation. When high-dose aspirin is discontinued, clinicians begin low-dose aspirin (3 to 5 mg/kg per day) and maintain it until the patient shows no evidence of coronary changes by 6 to 8 weeks after the onset of illness. After 6 to 8 weeks, aspirin is adjusted to a lower dose (2 to 3 mg/kg per day) until 6 months [4]

^b^: Clinicians begin low-dose aspirin (3 to 5 mg/kg per day) and maintain it until the patient shows no evidence of coronary changes by 6 to 8 weeks after the onset of illness. After 6 to 8 weeks, aspirin is adjusted to a lower dose (2 to 3 mg/kg per day) until 6 months [4]

^c^: 2 to 6 mg/kg per day in 3 divided doses [4]

### Parameters of echocardiography

The differences among the groups with respect to LV end-diastolic dimension, left atrial dimension, left atrial volumes, LV end-diastolic volume, LV end-systolic volume, stroke volume, LVEF, E wave, A wave, E/A, any tissue Doppler values, MAPSE, and TAPSE were not statistically significant (*P* > 0.05; Table 2).

**Table 2:** Parameter of echocardiography

	KD with CAD (*n* = 54)	KD without CAD (*n* = 46)	Controls (*n* = 51)	F	*P*
LVEDD (mm)	42.19 ± 3.43	41.46 ± 3.62	41.64 ± .3.47	0.600	0.551
LAD (mm)	23.57 ± 2.17	23.72 ± 2.81	24.05 ± 3.03	0.440	0.648
LAV_max_ (mm^3^)	29.64 ± 5.75	31.02 ± 5.64	29.40 ± 5.63	1.130	0.324
LAV_min_ (mm^3^)	11.38 ± 2.65	12.01 ± 2.61	11.27 ± 2.60	1.130	0.325
LVEDV (ml)	67.65 ± 15.93	68.08 ± 15.40	67.97 ± 19.32	0.015	0.992
LVESV (ml)	24.57 ± 7.40	24.61 ± 6.47	25.36 ± 8.21	0.188	0.836
SV (ml)	43.09 ± 9.58	43.46 ± 10.08	42.61 ± 12.35	0.089	0.926
LVEF (%)	64.02 ± 4.33	63.89 ± 4.12	62.64 ± 4.63	1.561	0.213
E wave (m/s)	1.01 ± 0.15	1.05 ± 0.13	1.06 ± 0.18	1.969	0.144
A wave (m/s)	0.59 ± 0.13	0.59 ± 0.12	0.60 ± 0.13	0.265	0.775
E/A	1.81 ± 0.52	1.85 ± 0.40	1.82 ± 0.38	0.124	0.890
Lateral s’ (cm/s)	8.49 ± 1.13	8.59 ± 0.93	8.34 ± 1.08	0.682	0.511
Lateral e’ (cm/s)	14.37 ± 2.42	15.10 ± 2.25	14.52 ± 2.38	1.331	0.267
Lateral a’ (cm/s)	6.33 ± 1.58	6.21 ± 1.27	6.49 ± 1.66	0.419	0.664
Lateral E/e’	7.13 ± 1.31	7.03 ± 1.08	7.25 ± 1.24	0.391	0.676
Lateral e’/a’	2.38 ± 0.59	2.50 ± 0.45	2.33 ± 0.49	1.264	0.288
Septal s’ (cm/s)	6.64 ± 1.96	7.24 ± 2.35	6.98 ± 2.19	0.993	0.376
Septal e’ (cm/s)	13.10 ± 2.95	13.44 ± 2.31	13.79 ± 2.52	0.897	0.413
Septal a’ (cm/s)	6.02 ± 1.55	5.68 ± 1.74	5.83 ± 2.31	0.394	0.677
Septal E/e’	8.21 ± 2.73	8.00 ± 1.67	8.04 ± 2.34	0.114	0.894
Septal e’/a’	2.34 ± 0.96	2.61 ± 1.01	3.01 ± 1.93	2.865	0.060
RV s’ (cm/s)	9.07 ± 2.70	9.28 ± 2.79	9.49 ± 2.85	0.301	0.741
RV e’ (cm/s)	15.72 ± 2.47	15.05 ± 2.77	15.72 ± 2.83	0.994	0.375
RV a’ (cm/s)	6.38 ± 1.57	6.48 ± 1.39	6.32 ± 1.55	0.142	0.867
RV e’/a’	2.62 ± 0.81	2.47 ± 0.87	2.71 ± 1.13	0.760	0.469
MAPSE	13.02 ± 1.74	13.09 ± 1.71	13.06 ± 1.78	0.021	0.979
TAPSE	20.32 ± 4.32	20.76 ± 3.68	20.22 ± 3.84	0.252	0.781

*CAD* coronary artery dilation; *LAD* left atrial dimension; *LAV* left atrial volume; *LVEDD* left ventricular end-diastolic dimension; *LVEDV* left ventricular end-diastolic volume; *LVEF* left ventricular ejection fraction; *LVESV* left ventricular end-systolic volume; *KD* Kawasaki disease; *MAPSE* mitral annular plane systolic excursion; *RV* right ventricle; *SV* stroke volume; *TAPSE* tricuspid annular plane systolic excursion

### LV myocardial strain using 2D STE

For regional strain, the differences among the groups with respect to basal anterior segment, basal anterolateral segment, mid-inferoseptal segment, mid-inferior segment, and all apical segments were not statistically significant (*P* > 0.05) (Table 3). Compared to the control group, the strain values regarding the basal inferoseptal segment, basal inferolateral segment, mid-anterior segment, mid-inferolateral segment, and mid-anterolateral segment decreased in the KD with CAD group but was unchanged in the KD without CAD group (*P* < 0.05). The strain values of the basal anteroseptal segment, basal inferior segment, and mid-anteroseptal segment decreased both in the KD with and without CAD groups, compared to the control group (*P* < 0.05).

**Table 3:** LV myocardial strain using 2D STE

2D STE	KD with CAD (*n* = 54)	KD without CAD (*n* = 46)	Controls (*n* = 51)	F	*P*
Regional					
Basal anterior (%)	−19.50 ± 9.24	− 21.72 ± 4.95	− 21.51 ± 4.03	1.785	0.171
Basal anteroseptal (%)	−19.80 ± 3.48*	− 19.24 ± 4.68#	− 21.75 ± 4.36	4.938	0.008
Basal inferoseptal (%)	−17.81 ± 2.60*	−18.33 ± 2.45	− 19.24 ± 3.01	3.691	0.027
Basal inferior (%)	−19.91 ± 3.45*	−19.91 ± 3.08#	−22.00 ± 3.54	6.482	0.002
Basal inferolateral (%)	−16.69 ± 6.80*	−17.78 ± 4.48	−19.88 ± 4.63	4.559	0.012
Basal anterolateral (%)	−17.65 ± 5.91	−20.04 ± 7.06	−19.94 ± 4.57	2.743	0.068
Mid anterior (%)	−21.24 ± 4.42*	− 21.93 ± 5.44	−23.73 ± 3.70	4.129	0.018
Mid anteroseptal (%)	−21.19 ± 4.01*	−21.02 ± 5.31#	− 23.06 ± 4.16	3.198	0.044
Mid inferoseptal (%)	−21.57 ± 2.46	−26.30 ± 29.61	−22.31 ± 2.66	1.154	0.318
Mid inferior (%)	−23.06 ± 4.27	−23.33 ± 3.02	− 23.49 ± 3.08	0.203	0.817
Mid inferolateral (%)	−18.48 ± 5.97*†	−20.54 ± 3.78	−21.02 ± 3.61	4.441	0.013
Mid anterolateral (%)	−19.11 ± 5.94*†	−21.28 ± 5.12	−21.75 ± 4.14	3.933	0.022
Apical anterior (%)	−20.81 ± 6.17	−21.78 ± 6.29	−22.53 ± 8.94	0.738	0.480
Apical septal (%)	−22.28 ± 4.04	−23.15 ± 4.56	− 23.47 ± 4.58	1.040	0.356
Apical inferior (%)	−22.91 ± 4.95	−24.20 ± 4.29	−23.65 ± 4.43	1.000	0.370
Apical lateral (%)	−20.37 ± 4.85	−22.04 ± 4.82	−22.37 ± 4.12	2.846	0.061
Apex (%)	−21.76 ± 4.12	−22.74 ± 4.43	−23.14 ± 4.20	1.463	0.235
Global					
GLS_LAX (%)	−18.81 ± 6.71*	−20.45 ± 3.18	−21.69 ± 2.72	5.059	0.007
GLS_A4C (%)	−19.80 ± 2.85*†	−21.20 ± 2.75	−21.28 ± 2.33	5.082	0.007
GLS_A2C (%)	−21.46 ± 3.00	−21.98 ± 2.84	−22.74 ± 2.75	2.616	0.075
2D GLS (%)	−20.29 ± 2.38*†	−21.20 ± 2.34	−21.84 ± 2.18	6.025	0.003

*KD with CAD group vs. control, *P* < 0.05; #KD without CAD group vs. control, *P* < 0.05; † KD with CAD group vs. KD without CAD group, *P* < 0.05. *CAD* coronary artery dilation; *GLS_A2C* global longitudinal strain_apical two-chamber; *GLS_A4C* Global longitudinal strain_apical four-chamber; *GLS_LAX* global longitudinal strain_long-axis; *KD* Kawasaki disease; *STE* speckle tacking echocardiography

The difference of 2D regional strain occurred in the mid-inferolateral segment and mid-anteroseptal segment which were assigned to the left circumflex coronary artery between the KD with and without CAD groups (*P* < 0.05) [21]. However, these decreasing had no involvement with left circumflex coronary artery (mid-inferolateral segment: *P* = 0.128; mid-anteroseptal segment: *P* = 0.249).

For global strain, the differences among the groups with GLS_A2C were not statistically significant (*P* > 0.05). Compared to the control group, GLS_LAX, GLS_A4C, and 2D GLS decreased in the KD with CAD group but was unchanged in the KD without CAD group (*P* < 0.05). There was a difference between males and females in the KD with CAD group, but no difference in the KD without CAD and control group (Supplement Table S1). The correlation between 2D GLS and the interval from onset to exam is shown in Supplement Table S2.

### LV myocardial strain using 3D STE

Compared to the control group, the GLS and GAS decreased in the KD with and without CAD groups (*P* < 0.05). GCS and GRS decreased in the KD with CAD group but was unchanged in the KD without CAD group (*P* < 0.05; Table 4). There was a difference between males and females in the KD with CAD group, but no difference in the KD without CAD and control group (Supplement Table S1). The correlation between 3D STE parameters and the interval from onset to exam were in Supplement Table S2.

**Table 4:** LV myocardial strain using 3D STE

3D STE	KD with CAD (*n* = 54)	KD without CAD (*n* = 46)	Controls (*n* = 51)	F	*P*
GLS (%)	−16.09 ± 3.00*†	−17.91 ± 4.50#	−19.84 ± 2.73	15.504	< 0.001
GCS (%)	−15.85 ± 3.31*†	−18.48 ± 7.20	−19.00 ± 3. 38	6.335	0.002
GRS (%)	45.46 ± 9.54*†	50.80 ± 14.29	53.73 ± 11.17	6.753	0.002
GAS (%)	−27.26 ± 4.51*	−28.11 ± 8.24#	−31.02 ± 4.50	5.775	0.004

*KD with CAD group vs. control, *P* < 0.05; #KD without CAD group vs. control, *P* < 0.05; † KD with CAD group vs. KD without CAD group, *P* < 0.05. *CAD* coronary artery dilation; *GLS* global longitudinal strain; *GCS* global circumferential strain; *GRS* global radial strain; *GAS* global area strain; *KD* Kawasaki disease; *STE* speckle tacking echocardiography

### ROC curve for the detection of LV dysfunction between KD with CAD and the controls

ROC curve analysis (Table 5) revealed that 3D GLS had a better ability to identify KD than 2D GLS, 3D GCS, 3D GRS, and 3D GAS (AUC = 0.819 vs. 0.684, 0.747, 0.712, and 0.717, respectively). The cut-off value for 3D GLS was − 17.50%.

**Table 5:** ROC curve for the detection of LV dysfunction between KD with CAD and the controls

	Cut-off (%)	AUC	Sensitivity (%)	Specificity (%)
2D GLS	−20.45	0.684	57.4	74.5
3D GLS	−17.50	0.819	70.4	78.4
3D GCS	−17.50	0.747	70.4	66.7
3D GRS	47.50	0.712	59.3	76.5
3D GAS	−27.50	0.717	55.6	76.5

*AUC* area under the curve; *CAD* coronary artery dilation; *GLS*: global longitudinal strain; *GCS* global circumferential strain; *GRS* global radial strain; *GAS* global area strain; *KD* Kawasaki disease; *STE* speckle tacking echocardiography

### Reproducibility

Inter- and intra-observer variability was good for all 3D global strains (Supplement Table S3).

## Discussion

The etiology of KD is unknown, but is more prevalent in Asians and Asian-Americans. Between 4000 and 10,000 new cases of KD are diagnosed each year in Americans and Asians [22, 23]. Although only 8.1% of patients have CALs in the acute phase, 32–50% of KD patients have coronary artery dimensions within the normal range, but with serial measurements demonstrate reductions in luminal dimensions suggestive of dilation using the patient as his or her own control, which may indicate that CAD may be more common than previously thought [14]. In the pre–IVIG era, coronary artery aneurysms occurred in 20 to 25% of KD patients in the acute phase [24]. The incidence of coronary artery aneurysms decreased to less than 5% after use of IVIG which became more widespread in the 1990s [25].

In the acute phase, KD not only induces vasculitis (causing CALs), but also affects the pericardium (causing pericardial effusions) [26], endocardium (causing valvulitis and valvar regurgitation) [26], and myocardium (causing myocarditis and systolic ventricular dysfunction) [10], as detected by echocardiography. A number of previous studies have focused on acute phase LV systolic dysfunction. LV myocardial strain obtained by STE in the acute phase is well-documented [5, 6, 8, 10]. Longitudinal strain is a more sensitive indicator of myocardial involvement in KD [5]. LV myocardial longitudinal strain is decreased at the onset of KD. Six-to-8 weeks after the timely administration of IVIG, myocardial strain, as an index of LV systolic function, has recovered [6, 8, 10]. When KD patients are subdivided into patients with and without CAD, both subgroups had similar longitudinal strain [5, 26]. This interesting result may indicate that CAD did not aggravate LV systolic dysfunction in the acute phase.

The Japanese Circulation Society concluded that cardiovascular symptoms in KD patients only appears two decades after the onset of the disease [27]. Thus, assessing LV systolic function in subclinical children with a history of KD, seems to be quite necessary. Few previous studies have focused on LV systolic function changes in patients with KD after long-term follow-up, although four studies assessed LV myocardial strain [28–31]. The results are conflicting. Compared to healthy children, Friesen et al. [28] reported a significant decrease in LV mid-anterior segment using cardiac magnetic resonance imaging (CMRI). Bratis et al. [29] showed no significant changes in GLS using CMRI. Dedeoglu et al. [30] demonstrated that a significant decrease of strain in the basal inferoseptal segment, basal anterolateral segment, apical septal segment, apical inferior segment, and GLS_A2C. No changes have been found in GLS_A4C or GLS_LAX. Yu et al. [31] used 3D STE and revealed a significant decrease in 3D GRS and 3D GAS. These four studies mainly focused on the effect of coronary artery aneurysms on LV myocardial strain. In addition, due to the small sample size in each study, subtle LV systolic dysfunction may not have been detected.

Echocardiography is a fast, noninvasive, and accurate imaging modality to diagnose KD. Meanwhile, follow-up using echocardiography is a convenient method for clinicians. The weakness of 2D STE is that different planes are obtained in different cardiac cycles, whereas 3D STE is able to obtain each plane in the same cardiac cycle. Thus, 3D STE is expected to be a more accurate and sensitive modality to detect subclinical myocardial dysfunction than 2D STE [32].

Our study assessed regional and global LV myocardial strain using 2D STE and 3D STE. For 2D regional strain, we found only the mid-inferolateral and mid-anteroseptal segments decreased in the KD with CAD group compared to the KD without CAD group. For 2D global strain, we found a significant decrease in GLS_A4C, GLS_LAX, and 2D GLS in the KD with CAD group compared to the controls; however, we did not find a difference between the KD without CAD group and the controls using 2D global strain. Then, 3D STE was performed. As might have been expected, we revealed that GLS and GAS decreased in KD without CAD group compared to the controls. We also found 3D GLS to be a better parameter (larger AUC) to detect LV systolic dysfunction than other 3D global strain, which showed that 3D GLS was gradually reduced in the three groups. The reason for the difference between the KD group and the controls is that myocarditis has been well-documented in 50–70% of KD patients in the acute phase with or without CALs [10, 14, 33]. KD-induced myocarditis may result in long-term sequelae [11, 12]. Persistent myocardial abnormalities (myocarditis and fibrosis) have been observed by autopsy and biopsy in children without coronary aneurysms even after 11 years from the onset of KD [11]. Second, KD makes the incidence of abnormal electrocardiograms (right axis deviation and incomplete right bundle-branch block) three times higher than normal high-school students [34]. The aforementioned mechanical and electrical disturbances due to KD could cause LV systolic dyssynchrony, which further leads to a decrease in LV systolic function [35]. Third, some previous studies revealed that myocardial blood flow and myocardial flow reserve were reduced in the KD with and without CAD group [36, 37]. This may be the reason why stain parameters of 2D SDE and 3D STE decreased in the KD with and without CAD groups, compared to the control group. Fourth, the previous study also revealed that the vasoconstriction and vasodilatation of coronary arteries were impaired in the KD with CAD group but unchanged in the KD without CAD group after the long-term onset of KD [38]. This coronary microvascular dysfunction was related to myocardial ischemia, as confirmed in previous studies [39, 40]. This means KD with CAD and KD without CAD groups may have different degrees of coronary microvascular dysfunction detected by several methods. It can explain why some stain parameters of 2D SDE and 3D STE decreased in the KD with CAD group, compared to the KD without CAD group.

This study had limitations. We did not include KD children with persistent or regressive coronary artery aneurysms. Therefore, the KD children without CAD cannot be compared with the persistent coronary artery aneurysm subgroup or regressive coronary artery aneurysm subgroup. We cannot further verify the hypothesis that the severity of CALs is positively correlated with the degree of LV systolic dysfunction.

## Conclusion

LV systolic dysfunction of KD children with CAD was shown to be more severe than KD children without CAD compared to healthy children. This dysfunction can be detected by 2D and 3D STE using LV regional and global myocardial strain.

## Supplementary information

**Additional file 1: Table S1.** Left ventricular myocardial strain using 2D and 3D STE. **Table S2.** Correlation between longitudinal strain values and the interval from onset to exam (months). **Table S3.** Reproducibility of 3D global strain.

## Data Availability

The datasets used and/or analyzed during the current study are available from the corresponding author on reasonable request.

## Abbreviations

CAD: Coronary artery dilatation
CAL: Coronary artery lesions
GAS: Global area strain
GCS: Global circumferential strain
GLS: Global longitudinal strain
GRS: Global radial strain
KD: Kawasaki disease
LVEF: Left ventricular ejection fraction
STE: Speckle tracking echocardiography

## References

[1] Bhatt M, Anil SR, Sivakumar K, Kumar K (2004). Neonatal Kawasaki disease. Indian J Pediatr.

[2] Makino N, Nakamura Y, Yashiro M, Ae R, Tsuboi S, Aoyama Y, Kojo T, Uehara R, Kotani K, Yanagawa H (2015). Descriptive epidemiology of Kawasaki disease in Japan, 2011-2012: from the results of the 22nd nationwide survey. J Epidemiol.

[3] Gordon JB, Kahn AM, Burns JC (2009). When children with Kawasaki disease grow up: myocardial and vascular complications in adulthood. J Am Coll Cardiol.

[4] Newburger JW, Takahashi M, Gerber MA, Gewitz MH, Tani LY, Burns JC, Shulman ST, Bolger AF, Ferrieri P, Baltimore RS (2004). Diagnosis, treatment, and long-term management of Kawasaki disease: a statement for health professionals from the committee on rheumatic fever, endocarditis and Kawasaki disease, council on cardiovascular disease in the young, American Heart Association. Circulation.

[5] McCandless RT, Minich LL, Wilkinson SE, McFadden ML, Tani LY, Menon SC (2013). Myocardial strain and strain rate in Kawasaki disease. Eur Heart J Cardiovasc Imaging.

[6] Xu QQ, Ding YY, Lv HT, Zhou WP, Sun L, Huang J, Yan WH (2014). Evaluation of left ventricular systolic strain in children with Kawasaki disease. Pediatr Cardiol.

[7] Hematian MN, Torabi S, MalaKan-Rad E, Sayadpour-Zanjani K, Ziaee V, Lotfi-Tolkaldany M (2015). Noninvasive evaluation of myocardial systolic dysfunction in the early stage of Kawasaki disease: a speckle-tracking echocardiography study. Iran J Pediatr.

[8] Azak E, Cetin II, Gursu HA, Kibar AE, Surucu M, Orgun A, Pamuk U (2018). Recovery of myocardial mechanics in Kawasaki disease demonstrated by speckle tracking and tissue Doppler methods. Echocardiography.

[9] Wang H, Shang J, Tong M, Song Y, Ruan L (2019). Evaluation of left ventricular function in immunoglobulin-resistant children with Kawasaki disease: a two-dimensional speckle tracking echocardiography study. Clin Cardiol.

[10] Yu JJ, Choi HS, Kim YB, Son JS, Kim YH, Ko JK, Park IS (2010). Analyses of left ventricular myocardial deformation by speckle-tracking imaging during the acute phase of Kawasaki disease. Pediatr Cardiol.

[11] Yutani C, Go S, Kamiya T, Hirose O, Misawa H, Maeda H, Kozuka T, Onishi S (1981). Cardiac biopsy of Kawasaki disease. Arch Pathol Lab Med.

[12] Liu AM, Ghazizadeh M, Onouchi Z, Asano G (1999). Ultrastructural characteristics of myocardial and coronary microvascular lesions in Kawasaki disease. Microvasc Res.

[13] Mitani Y, Okuda Y, Shimpo H, Uchida F, Hamanaka K, Aoki K, Sakurai M (1997). Impaired endothelial function in epicardial coronary arteries after Kawasaki disease. Circulation.

[14] McCrindle BW, Rowley AH, Newburger JW, Burns JC, Bolger AF, Gewitz M, Baker AL, Jackson MA, Takahashi M, Shah PB (2017). Diagnosis, treatment, and long-term Management of Kawasaki Disease: a scientific statement for health professionals from the American Heart Association. Circulation.

[15] Ghelani SJ, Brown DW, Kuebler JD, Perrin D, Shakti D, Williams DN, Marx GR, Colan SD, Geva T, Harrild DM (2018). Left atrial volumes and strain in healthy children measured by three-dimensional echocardiography: Normal values and maturational changes. J Am Soc Echocardiogr.

[16] Matos J, Kronzon I, Panagopoulos G, Perk G (2012). Mitral annular plane systolic excursion as a surrogate for left ventricular ejection fraction. J Am Soc Echocardiogr.

[17] Ghio S, Recusani F, Klersy C, Sebastiani R, Laudisa ML, Campana C, Gavazzi A, Tavazzi L (2000). Prognostic usefulness of the tricuspid annular plane systolic excursion in patients with congestive heart failure secondary to idiopathic or ischemic dilated cardiomyopathy. Am J Cardiol.

[18] Koestenberger M, Ravekes W, Everett AD, Stueger HP, Heinzl B, Gamillscheg A, Cvirn G, Boysen A, Fandl A, Nagel B (2009). Right ventricular function in infants, children and adolescents: reference values of the tricuspid annular plane systolic excursion (TAPSE) in 640 healthy patients and calculation of z score values. J Am Soc Echocardiogr.

[19] Voigt JU, Pedrizzetti G, Lysyansky P, Marwick TH, Houle H, Baumann R, Pedri S, Ito Y, Abe Y, Metz S (2015). Definitions for a common standard for 2D speckle tracking echocardiography: consensus document of the EACVI/ASE/industry task force to standardize deformation imaging. J Am Soc Echocardiogr.

[20] Luis SA, Yamada A, Khandheria BK, Speranza V, Benjamin A, Ischenko M, Platts DG, Hamilton-Craig CR, Haseler L, Burstow D (2014). Use of three-dimensional speckle-tracking echocardiography for quantitative assessment of global left ventricular function: a comparative study to three-dimensional echocardiography. J Am Soc Echocardiogr.

[21] Cerqueira MD, Weissman NJ, Dilsizian V, Jacobs AK, Kaul S, Laskey WK, Pennell DJ, Rumberger JA, Ryan T, Verani MS (2002). Standardized myocardial segmentation and nomenclature for tomographic imaging of the heart. A statement for healthcare professionals from the cardiac imaging Committee of the Council on clinical cardiology of the American Heart Association. Circulation.

[22] Holman RC, Curns AT, Belay ED, Steiner CA, Schonberger LB (2003). Kawasaki syndrome hospitalizations in the United States, 1997 and 2000. Pediatrics.

[23] Nakamura Y, Yashiro M, Uehara R, Oki I, Kayaba K, Yanagawa H (2008). Increasing incidence of Kawasaki disease in Japan: nationwide survey. Pediatr Int.

[24] Kato H, Sugimura T, Akagi T, Sato N, Hashino K, Maeno Y, Kazue T, Eto G, Yamakawa R (1996). Long-term consequences of Kawasaki disease. A 10- to 21-year follow-up study of 594 patients. Circulation.

[25] Newburger JW (1996). Treatment of Kawasaki disease. Lancet.

[26] Gaur L, Waloff K, Schiller O, Sable CA, Frank LH (2014). Noncoronary inflammation in Kawasaki disease is associated with abnormal myocardial deformation in the acute phase. J Am Soc Echocardiogr.

[27] Group JCSJW (2014). Guidelines for diagnosis and management of cardiovascular sequelae in Kawasaki disease (JCS 2013). Digest version. Circ J.

[28] Friesen RM, Schafer M, Jone PN, Appiawiah N, Vargas D, Fonseca B, MV DM, Truong U, Malone L, Browne LP (2017). Myocardial perfusion reserve index in children with Kawasaki disease. J Magn Reson Imaging.

[29] Bratis K, Hachmann P, Child N, Krasemann T, Hussain T, Mavrogeni S, Botnar R, Razavi R, Greil G (2017). Cardiac magnetic resonance feature tracking in Kawasaki disease convalescence. Ann Pediatr Cardiol.

[30] Dedeoglu R, Barut K, Oztunc F, Atik S, Adrovic A, Sahin S, Cengiz D, Kasapcopur O (2017). Evaluation of myocardial deformation in patients with Kawasaki disease using speckle-tracking echocardiography during mid-term follow-up. Cardiol Young.

[31] Yu W, Wong SJ, Cheung YF: Left ventricular mechanics in adolescents and young adults with a history of kawasaki disease: analysis by threedimensional speckle tracking echocardiography. Echocardiography 2014; 31(4):483-491. 10.1111/echo.12394. 10.1111/echo.12394 24804605

[32] Geyer H, Caracciolo G, Abe H, Wilansky S, Carerj S, Gentile F, Nesser HJ, Khandheria B, Narula J, Sengupta PP (2010). Assessment of myocardial mechanics using speckle tracking echocardiography: fundamentals and clinical applications. J Am Soc Echocardiogr.

[33] Dionne A, Dahdah N (2018). Myocarditis and Kawasaki disease. Int J Rheum Dis.

[34] Hirata S, Nakamura Y, Matsumoto K, Yanagawa H (2002). Long-term consequences of Kawasaki disease among first-year junior high school students. Arch Pediatr Adolesc Med.

[35] Yu Y, Sun K, Xue H, Chen S, Yang J (2013). Usefulness of real-time 3-dimensional echocardiography to identify and quantify left ventricular dyssynchrony in patients with Kawasaki disease. J Ultrasound Med.

[36] Muzik O, Paridon SM, Singh TP, Morrow WR, Dayanikli F, Di Carli MF (1996). Quantification of myocardial blood flow and flow reserve in children with a history of Kawasaki disease and normal coronary arteries using positron emission tomography. J Am Coll Cardiol.

[37] Furuyama H, Odagawa Y, Katoh C, Iwado Y, Ito Y, Noriyasu K, Mabuchi M, Yoshinaga K, Kuge Y, Kobayashi K (2003). Altered myocardial flow reserve and endothelial function late after Kawasaki disease. J Pediatr.

[38] Iemura M, Ishii M, Sugimura T, Akagi T, Kato H (2000). Long term consequences of regressed coronary aneurysms after Kawasaki disease: vascular wall morphology and function. Heart.

[39] Ong P, Athanasiadis A, Mahrholdt H, Borgulya G, Sechtem U, Kaski JC (2012). Increased coronary vasoconstrictor response to acetylcholine in women with chest pain and normal coronary arteriograms (cardiac syndrome X). Clin Res Cardiol.

[40] Matsuda K, Teragawa H, Fukuda Y, Ueda K, Higashi Y, Sakai K, Miura F, Hirao H, Yamagata T, Yoshizumi M (2003). Response of the left anterior descending coronary artery to acetylcholine in patients with chest pain and angiographically normal coronary arteries. Am J Cardiol.

