# Supplementary material for: Assessing left ventricular systolic function in children with a history of Kawasaki disease
Source: BMC Cardiovasc Disord. 2020 Mar 12;20:131. doi: 10.1186/s12872-020-01409-0 (PMC7068877; doi:10.1186/s12872-020-01409-0)
Supplement: Supplementary file 1 — Additional file 1: Table S1. Left ventricular myocardial strain using 2D and 3D STE. Table S2. Correlation between longitudinal strain values and the interval from onset to exam (months). Table S3. Reproducibility of 3D global strain. [file 12872_2020_1409_MOESM1_ESM.docx]

**Table S1. Left ventricular myocardial strain using 2D and 3D STE**

|  | Male | Female | *t* | *P* |
| --- | --- | --- | --- | --- |
| KD with CAD |  |  |  |  |
|  | 41 | 13 |  |  |
| 2D GLS | -19.88±2.42 | -21.62±1.71 | 2.398 | 0.020 |
| 3D GLS | -16.20±3.02 | -18.23±2.83 | 2.149 | 0.036 |
| 3D GCS | -15.71±3.29 | -16.31±3.47 | 0.566 | 0.574 |
| 3D GAS | -27.10±4.09 | -27.77±5.81 | 0.465 | 0.644 |
| 3D GRS | 44.63±9.22 | 48.08±10.42 | -1.137 | 0.261 |
| KD without CAD |  |  |  |  |
|  | 29 | 17 |  |  |
| 2D GLS | -21.55±2.05 | -20.65±2.71 | -1.281 | 0.207 |
| 3D GLS | -18.21±4.10 | -17.41±5.21 | -0.574 | 0.569 |
| 3D GCS | -19.14±8.13 | -17.35±5.31 | -0.808 | 0.423 |
| 3D GAS | -27.76±8.82 | -28.71±7.36 | 0.373 | 0.711 |
| 3D GRS | 50.86±12.04 | 50.71±17.90 | 0.035 | 0.972 |
| Controls |  |  |  |  |
|  | 34 | 17 |  |  |
| 2D GLS | -21.97±2.23 | -21.40±2.46 | -0.838 | 0.406 |
| 3D GLS | -19.48±2.53 | -20.75±3.31 | 1.366 | 0.179 |
| 3D GCS | -18.61±2.68 | -21.08±4.21 | 1.903 | 0.077 |
| 3D GAS | -30.48±3.78 | -32.58±6.05 | 1.393 | 0.171 |
| 3D GRS | 52.21±8.59 | 58.50±15.52 | -1.331 | 0.205 |

CAD: coronary artery dilation; GLS: global longitudinal strain; GCS: global circumferential strain; GRS: global radial strain; GAS: global area strain; KD: Kawasaki disease; STE: speckle tacking echocardiography.

**Table S2 Correlation between longitudinal strain values and**

**the interval from onset to exam (months)**

|  | KD with CAD (n = 54) | | KD without CAD (n = 46) | | KD (n = 100) | |
| --- | --- | --- | --- | --- | --- | --- |
|  | *r* | *P* | *r* | *P* | *r* | *P* |
| 2D GLS | 0.317 | 0.019 | 0.135 | 0.372 | 0.217 | 0.030 |
| 3D GLS | 0.100 | 0.471 | 0.452 | 0.002 | 0.292 | 0.003 |
| 3D GCS | 0.002 | 0.987 | 0.163 | 0.279 | 0.097 | 0.337 |
| 3D GAS | 0.254 | 0.064 | -0.033 | 0.826 | 0.065 | 0.517 |
| 3D GRS | -0.213 | 0.122 | -0.249 | 0.095 | -0.220 | 0.028 |

CAD: coronary artery dilation; GLS: global longitudinal strain; GCS: global circumferential strain; GRS: global radial strain; GAS: global area strain; KD: Kawasaki disease.

**Table S3. Reproducibility of 3D global strain**

|  |  | Inter-observer | |  | Intra-observer | |
| --- | --- | --- | --- | --- | --- | --- |
|  |  | ICC | CoV (%) |  | ICC | CoV (%) |
| KD with CAD | GLS | 0.96 (0.73, 0.98) | 9.4 |  | 0.88(0.51, 0.97) | 7.7 |
|  | GCS | 0.85 (0.40, 0.96) | 4.1 |  | 0.87 (0.49, 0.97) | 7.7 |
|  | GRS | 0.88 (0.50, 0.97) | 11.2 |  | 0.86 (0.43, 0.97) | 5.7 |
|  | GAS | 0.89 (0.56, 0.97) | 3.9 |  | 0.88 (0.50, 0.97) | 5.5 |
| KD without CAD | GLS | 0.93 (0.70, 0.98) | 8.6 |  | 0.89 (0.54, 0.97) | 6.7 |
|  | GCS | 0.89 (0.57, 0.97) | 4.4 |  | 0.88 (0.51, 0.97) | 10.5 |
|  | GRS | 0.92 (0.69, 0.98) | 10.5 |  | 0.88 (0.53, 0.97) | 6.7 |
|  | GAS | 0.85 (0.39, 0.96) | 2.5 |  | 0.92 (0.67, 0.98) | 2.4 |

CAD: coronary artery dilation; CoV: coefficient of variance; GLS: global longitudinal strain; GCS: global circumferential strain; GRS: global radial strain; GAS: global area strain; ICC: intraclass correlation coefficient; KD: Kawasaki disease.
